# Concept flavor names from English language ENDS advertising in the USA, 2015–2020

**DOI:** 10.18332/tid/205762

**Published:** 2025-08-28

**Authors:** Ryan David Kennedy, Lauren Czaplicki, Meghan Bridgid Moran

**Affiliations:** 1 Department of Health, Behavior, and Society, Johns Hopkins Bloomberg School of Public Health, Baltimore, United States; 2 Institute for Global Tobacco Control, Johns Hopkins Bloomberg School of Public Health, Baltimore, United States

**Keywords:** tobacco products, tobacco industry, marketing, flavor

## Abstract

**INTRODUCTION:**

Some manufacturers of electronic nicotine delivery systems (ENDS) and liquids sell products with flavor names that use ambiguous terminology rather than explicitly characterize the product’s flavor. These are often referred to as ‘concept flavors’. This article presents a sample of ENDS concept flavor names to identify naming conventions the industry uses.

**METHODS:**

We reviewed 4546 English-language ENDS advertisements that ran in a variety of outlets including magazines, TV, radio, and direct-to-consumer emails, during the period 2015–2020. ENDS flavors in ads were identified and classified as ‘concept’ if the name contained no explicit characterizing flavor information. All concept flavor names were then reviewed by two coders to identify themes and practices used by ENDS manufacturers.

**RESULTS:**

The sample identified 215 unique concept flavor names. We found examples of flavor names that were suggestive of food or drink such as ‘Citra Zing’, as well as flavor names suggestive of sensations associated with drug use, such as ‘Blurred Vision’. Some other concept flavor themes identified included the use of colors (‘Red Venom’), numbers (‘No 42.’), words associated with mythology (‘God Nectar’), tropical imagery (Hawaiian Rainbow), and pop culture (‘Miami Vice’). Some naming practices included the creation of flavor names using a portmanteau (‘Grappleberry’), flavor names that used intentional misspelling or satiric misspelling, (‘Gritz’, and ‘Cap’n Crunk’), and the use of malapropisms (‘ohm sweet ohm’).

**CONCLUSIONS:**

The tobacco industry uses concept flavor names, and these names are presented in advertising in the US. This article identified themes and naming practices for concept flavors. Future work may continue to monitor the use of concept flavors and if concept flavor names are used in response to flavor restrictions. Additionally, future research may explore if concept flavors themes are associated with perceived risk, use intentions and behavior.

## INTRODUCTION

Almost all electronic nicotine delivery systems (ENDS) and liquids sold in the United States are flavored^1^, and the number of unique ENDS flavors available in the marketplace is ever changing. Ma et al.^2^ conducted a search of online ENDS retailers in the US in 2021 and identified over 14000 flavored e-liquid products. ENDS flavor names are typically delineated in the literature as either a characterizing flavor (e.g. Cherry, Vanilla), which clearly describe the product flavor, or as a concept flavors (e.g. Rainbow, Fusion) which have been commonly defined as flavor names that are vague/ambiguous and do not characterize the way the product tastes^3^. Kreslake et al.^4^ more broadly define concept flavors names as those that may evoke a sensory experience, including senses beyond taste such as ‘Red Hot’, as thermal perceptions are generally considered a component of the sense of touch^4^.

There have been various restrictions on the sale of flavored ENDS products at both federal and local levels. Federally, starting in February 2020 the U.S. Food & Drug Administration (FDA) has prioritized enforcement against the sale of rechargeable cartridge-based ENDS in characterizing flavors other than tobacco and menthol that do not have premarket authorization. Local restrictions include Minneapolis and St. Paul, Minnesota’s 2016 implementation of a flavored tobacco products restriction, which included ENDS products^5^. However, the tobacco industry has found ways to continue to sell flavored products, despite restrictions. For example, FDA’s enforcement prioritization did not apply to flavored disposable ENDS, and in December 2022, the CDC reported that sales of flavored disposable ENDS products increased. Moreover, this enforcement prioritization did not explicitly include non-characterizing flavors such as concept flavors but rather prioritized any ENDS product that is targeted to minors and 5.6% of product sales were for devices with an ‘ambiguous or concept flavor’^6^.

Cigar manufacturers have used concept flavors for years, possibly to circumvent state and local flavored tobacco bans^7^. ENDS manufacturer Bidi Stick changed flavor names that previously had unambiguous characterizing flavors like ‘fruity mango’ to noncharacterizing, concept flavor names like ‘Gold’^8^.

Product flavors are often featured in ENDS ads^9^. As such, there is value in further refining the existing typology of concept flavor names to provide insight into industry naming practices, so we may better understand how these naming practices influence perceptions of ENDS products, as well as intentions to use and actual use of ENDS. However, typologies of concept flavor names vary greatly^10,11^, making it difficult to compare findings across studies.

The present study qualitatively reviewed concept flavor names present in a sample of English language ENDS advertisements and identified different themes and naming practices used to derive concept flavor names.

## METHODS

### Sample

The study used English-language ENDS ads purchased from Numerator, a firm that monitors and collects advertisements from multiple channels including magazines, radio, TV, online and direct email. The sample included all ads identified by Numerator (n=4546) that ran during 2015 to 2020.

### Flavor name classification

Details about the coding process are reported in detail elsewhere^9^, but briefly each ad was reviewed and ENDS flavor names in the ad were recorded and coded as ‘concept’ if the name had no obvious characterizing flavor. We set a reliability standard of 0.80; inter-rater reliability among the coders exceeded this standard. Any discrepancies were reconciled via review to achieve consensus. This sample of ‘concept flavor’ names were subsequently reviewed by two coders to characterize themes or naming practices identifiable among the sample. Two a priori codes were used: 1) flavor names that were suggestive of food or drink, and 2) flavor names that were suggestive of tobacco/nicotine products (including mentholated products) and/or other drug use. Emergent themes and practices were iteratively defined through a collaborative process between the two coders. Each coder identified emergent themes independently, these were then discussed, and themes were added to the coding process. After each round of theme identification, the sample was re-coded until the majority of concept flavors were classified. Any discrepancies were reconciled by the coders. The themes and naming practices identified are presented below with representative example concept flavor names.

## RESULTS

### Sample

The study identified 215 unique concept flavors (Table 1). Some concept flavor names included a word that could be characterizing, but the entirety of the flavor name was nonsensical, such as the flavor ‘meteor milk’. In this example, ‘milk’ is a characterizing flavor, but ‘meteor milk’ is ambiguous. Concept flavors could be coded under more than one classification.

**Table 1:** Concept flavor names by theme and name construction practices

*Theme*	*Concept flavor name*
**Suggestive of food or drink**	‘Blue Ade’, ‘Brazzy’, ‘Cap’n Crunk’, ‘Citra Zing’, ‘Cloud Punch’, ‘Cob Roller’, ‘Dapple Whip’, ‘Farley’s Gnarly Sauce’, ‘Fosterz’, ‘God’s Nectar’, ‘Grappleberry’, ‘Gritz’, ‘Hawaiian POG’, ‘Honey Moon’, ‘Kande’, ‘Kande Ice’, ‘Krunchy Squares’, ‘Lahaina Lilikoi’, ‘Lava Flow Ice’, ‘Lush Ice’, ‘Mauna Dew’, ‘Meteor Milk’, ‘Mystery Pop’, ‘Nilla Nade’, ‘Ohmgurt’, ‘POG’, ‘Purple Ade’, ‘Rainbow Drop’, ‘Red Ade’, ‘Red Hot’, ‘Refresh’, ‘Refreshing’, ‘Smurf Sauce’, ‘Sow Sauce’, ‘Switch Sauce’, ‘The Razz’, ‘VSOP’, ‘Wi-Go-Nana’
**Suggestive of tobacco/nicotine products/mentholated products or other drug use**	‘Arctic Cloud’, ‘Blurred Vision’, ‘Brain Freeze’, ‘Carnal Ecstasy’, ‘Fosterz’, ‘Hedon’s bite’, ‘Kande Ice’, ‘Kool Lagerfeld’, ‘Lava Flow Ice’, ‘Lush Ice’, ‘Night Shade’, ‘Nightcap’, ‘Ohm Sweet Ohm’, ‘Ohmgurt’, ‘Ohmother’, ‘Polar Breeze’, ‘Popskull’, ‘Satisfying’, ‘Very Cool’, ‘VSOP’, ‘Winter Solstice’
**Include numbers or colors**	‘Beta’, ‘Black Mamba’, ‘Blackthorn’, ‘Blu Dream’, ‘Blu RZA Thunderbomb’, ‘Blue’, ‘Blue Ade’, ‘Blue Blood’, ‘Blue Magic’, ‘Bluetiful Disaster’, ‘Chester White’, ‘Fool’s Gold’, ‘Gold’, ‘Green’, ‘Green Blast’, ‘Hunnid K’, ‘Liquid Gold’, ‘LJ4’, ‘Neon Dream’, ‘No. 42’, ‘No. 51’, ‘Organic Green Goblin’, ‘Pink’, ‘Pink Burst’, ‘Pinky’, ‘Platinum’, ‘Pot O’Gold’, ‘Purple Ade’, ‘Purple Powder’, ‘Purple Reign’, ‘Rainbow Drop’, ‘Rainbow Riot’, ‘Red’, ‘Red Ade’, ‘Red Hot’, ‘Red Hot Lava’, ‘Red October’, ‘Red Venom’, ‘Royalty II’, ‘Ruby’, ‘Tan’
**Terminology associated with celestial bodies and/or mythological/religious figures/events**	‘Andromeda’, ‘Arctic Cloud’, ‘Astro’, ‘Babyclouds’, ‘Bossa Nova’, ‘Cerberus’, ‘Cloud Punch’, ‘Dragon Scape’, ‘Dragonaire’, ‘Dragonglass’, ‘God’s Nectar’, ‘Gryphon’, ‘Honey Moon’, ‘Maui Sun’, ‘Meteor Milk’, ‘Minotaur’, ‘Naked Unicorn’, ‘Organic Green Goblin’, ‘Parsec’, ‘Phoenix’, ‘Pluto’, ‘Pulsar’, ‘Rainbow Drop’, ‘Rainbow Riot’, ‘Rapture’, ‘Ryu’, ‘Sacré Coeur’, ‘Samba Sun’, ‘Snap Dragon’, ‘Sonrise’, ‘Voodoo’
**Terminology associated with vacation settings**	‘Bali Paradise’, ‘Beachy’, ‘Breezy’, ‘Caribbean Oasis’, ‘Hawaiian POG’, ‘Island Breeze’, ‘Island Squeeze’, ‘Lahaina Lilikoi’, ‘Maui Sun’, ‘Maui Waui’ ‘Mauna Dew’, ‘Miami Vice’, ‘Ocean Blast’, ‘Sea King’, ‘Summer Fusion’, ‘Tan’, ‘Tropics’, ‘Wet and Wavy’
**Terminology associated with places**	‘Jungle Fever’, ‘Lantern’s Keep’, ‘Open Road’, ‘Polar Breeze’, ‘Roadhouse’, ‘Sahara’, ‘Yamoto’
**Terminology associated with violence or destruction**	‘Fire Blast’, ‘Green Blast’, ‘Grimsvotn’, ‘Hedonsꞌ Bite’, ‘Lava Flow’, ‘Lava Flow Ice’, ‘Loop Ninja’, ‘Ocean Blast’, ‘Red Hot Lava’, ‘Red Venom’, ‘Slash’, ‘Sudden Death’, ‘Sweet Lava’, ‘Toxic’, ‘Vesuvius’, ‘Weaponized AK-12’
**Terminology associated with sex**	‘Carnal Ecstasy’, ‘G-string’, ‘Lush’, ‘Lush Ice’, ‘Nightlife’, ‘Orgasm’, ‘Para Mour’, ‘Siren’
**Terminology associated with people/identity/personalities and pop culture**	‘Balanced’, ‘Birthday Bash’, ‘Blue Blood’, ‘Bossa Nova’, ‘Cap’n Crunk’, ‘Capone’, ‘Clubs’, ‘Daydreamer’, ‘Diamonds’, ‘Don’t Care Bear’, ‘Don Juan Reserve’, ‘Duchess Reserve’, ‘Fool’s Gold’, ‘Frozen Hulk Tears’, ‘Gameover’, ‘Gone Baby Gone’, ‘Harlequin’, ‘Hearts’, ‘Hot Mess’, ‘Kool Lagerfeld’, ‘Lola’, ‘Loop Ninja’, ‘Lucky Bastard’, ‘Magic Man’, ‘Miami Vice’, ‘Napoleon’s Fave’, ‘Nona’, ‘Organic Green Goblin’, ‘Pan-Am’, ‘Pluto’, ‘Quinn’, ‘Red October’, ‘Roper’, ‘Royalty II’, ‘Samba Sun’, ‘Scofflaw’, ‘Sea King’, ‘Smurf Sauce’, ‘Sonic’, ‘Spades’, ‘Swag’, ‘Swagger’, ‘Tango Chillin’, ‘The Carter’, ‘The Man’, ‘The One’
**Terminology associated with animals**	‘Bird Brains’, ‘Black Mamba’, ‘Diamond Head’, ‘Don’t Care Bear’, ‘Roar’, ‘Snake Oil’, ‘Wacky Pony’, ‘Wonder Worm’
**Terminology associated with science/technology**	‘Beta’, ‘Chaos Theory’, ‘Fusion’, ‘Horsepower’, ‘Ohm Sweet Ohm’, ‘Ohmgurt’, ‘Ohmother’, ‘Metronome’, ‘Sonic’
**Name construction practices**	
**Portmanteaux**	‘Bluetiful Disaster’, ‘Grappleberry’, ‘Ohmgurt’, ‘Ohmother’, ‘Blu RZA Thunderbomb’
**Intentional misspelling or satiric misspelling**	‘Babyclouds’, ‘Blu Dream’, ‘Blu RZA Thunderbomb’, ‘Brazzy’, ‘Cap’n Crunk’, ‘Citra Zing’, ‘Dabble Dooyah’, ‘Do-Ragg’, ‘Dragonaire’, ‘Ez Duz It’, ‘Fosterz’, ‘Gameover’, ‘Got’eem!’, ‘Hunnid K’, ‘Kande’, ‘Kande Ice’, ‘Kool Lagerfeld’, ‘Krunchy Squares’, ‘Kryp’, ‘Maui Waui’, ‘Night Shade’, ‘Para Mour’, ‘Sonrise’, ‘Tango Chillin’, ‘The Razz’, ‘Wi-Go-Nana’
**Malapropism**	‘Ohm Sweet Ohm’, ‘Purple Reign’
**Not classified**	‘?Mystery?’, ‘Airmail’, ‘Breaker’, ‘Congress’, ‘Day Break’, ‘Delight’, ‘Froopy’, ‘Indulge’, ‘Keen’, ‘More Please’, ‘Pure’, ‘Rise’, ‘Rock’, ‘Sutro’, ‘Sublime’, ‘Tempting’, ‘The Berg’, ‘The Shape’, ‘Underwood’, ‘Unichew’, ‘Unleashed’

### Concept flavor names suggestive of food or drink

Our sample included flavor names that were suggestive of food or drink. Some concept flavors sounded very similar to characterizing foods/drink such as ‘Citra Zing’, which is close to the word citrus, and ‘Gritz’, which is close to the word ‘grits’ (a type of porridge). The concept flavor ‘Mauna Dew’ is like the soda ‘Mountain Dew’ and is possibly referring to Hawaiian term for mountain, Mauna. There were concept flavors that used the term ‘sauce’, including ‘Farley’s Gnarley sauce’, ‘Sow Sauce’, and ‘Smurf Sauce’, which are each ambiguous. Several concept flavors were a play on lemonade including ‘Nilla Nade’ which is a play on vanilla and lemonade, as well as ‘Blue Ade’, ‘Purple Ade’ and ‘Red Ade’.

### Concept flavor names suggestive of tobacco/nicotine products (including mentholated products) or other drug use

Some concept flavor names were suggestive of tobacco, such as ‘Night Shade’, which is likely a reference to the family of plants tobacco is a member (nightshade, genus Solanum). One concept flavor, ‘Kool Lagerfeld’ seems to be a play on the fashion designer Karl Lagerfeld, but Kool is spelled in the same way the Imperial Tobacco brand ‘Kool’ is spelled. Kool also a misspelling of ‘cool’ that could suggest a mentholated product. The concept flavor ‘Nightcap’, in addition to being a cap worn with nightclothes, is a type of alcoholic drink taken at bedtime or at the end of a festive evening. One concept flavor, ‘Blurred Vision’ is suggestive of sensations one might experience using drugs. Several concept flavors used terminology associated with cooling sensations such as ‘Lush Ice’, ‘Kande Ice’, ‘Polar Breeze’, ‘Very Cool’, and ‘Winter Solstice’, which are suggestive of menthol or mint products.

### Concept flavor names that include numbers or colors

Some concept flavors used numbers with ambiguous flavors including ‘LJ4’. Other concept flavors, ‘No 42.’ And ‘No. 51.’ have names similar to perfumes (e.g. Chanel No. 5). Colors were commonly used in concept flavor names, including, ‘Blackthorn’, ‘Blue Magic’, ‘Green Blast’, ‘Neon Dream’. Red was the most common color used in concept flavors with names such as, ‘Red Hot Lava’ and ‘Red Venom’.

### Concept flavor names that include terminology associated with celestial bodies and/or mythological/religious figures and events

The sample included concept flavor names that were classified as celestial including ‘Andromeda’, and ‘Astro’. Some concept flavor names included the word ‘cloud’, like ‘Babyclouds’ or ‘Arctic cloud’. The concept flavor name ‘Cloud Punch’ could be celestial and/or suggestive of drink, as punch is a drink as well as a violent act. The flavor ‘Pluto’ could be celestial (planet or planetoid) or mythological (Roman God), or possibly a reference to the cartoon character. Other concept flavor names that were classified as mythological included ‘Dragon Scape’, ‘Gryphon’, and ‘Minotaur’. Some terms used could be associated with religious events or terms, such as ‘Rapture’ and ‘Sacré Coeur’.

Names with ‘cloud’ may also refer to the pop culture act of ‘cloud chasing’, the phenomenon where ENDS users compete or showcase the creation of a large cloud of aerosol^12^.

### Concept flavor names that include terminology associated with vacations and/or vacation settings

These names often identified a place/region associated with vacations, including ‘Bali Paradise’, ‘Caribbean Oasis’, ‘Hawaiian POG’. Others were more generic and referred to general vacation or tropical settings such as ‘Island Breeze’, ‘Beachy’, and ‘Ocean Blast’.

### Concept flavor names that include terminology associated with places

Some flavor names were identified were generic places, such as ‘Roadhouse’, or ‘Open Road’. One flavor, ‘Lantern’s Keep’ could refer to a keep in a castle (secure place).

### Concept flavor names that include terminology associated with violence or destruction

Some concept flavor names were classified as having names that were violent or destructive including ‘Slash’ and ‘Weaponized AK-12’. One concept flavor, ‘Grimsvotn’ is the same name as an active volcano in Iceland. The word ‘lava’ was included in concept flavor names including ‘Lava Flow’.

### Concept flavor names that include terminology associated with sex

Some concept flavor names were classified as suggestive of or associated with sex, including ‘Carnal Ecstasy’, ‘Orgasm’, and ‘G-string’.

### Concept flavor names that include terminology associated with people/identity/personalities and pop culture

Some concept flavors aligned with people/identities/personalities such as, ‘Magic Man’, ‘Hot Mess’, ‘Lucky Bastard’, ‘Daydreamer’, and ‘the Man’. The concept flavor ‘Harlequin’ could refer to a mute character in traditional pantomime/Commedia Dell’arte or be a reference to the character in DC comics/movies. Other concept flavor names were direct references to pop culture figures, such as ‘Don’t Care Bear’, ‘Cap’n Crunk’, or historical figures such as ‘Napoleon’s Fave’. Other flavor names were people’s names, such as ‘Lola’, which could just be a name, or it could be referring to the song ‘Lola’ by the Kinks. There were some concept flavors that incorporated words associated with royalty including ‘Sea King’, ‘Duchess Reserve’, and ‘Royalty II’. Some flavor names were classified as direct reference to popular culture including ‘Miami Vice’ (TV show and movie). The flavor ‘Red October’ could be referring to the 1990 movie ‘The Hunt for Red October’.

### Concept flavor names that include terminology associated with animals

Names of animals were incorporated into some concept flavor names including ‘Bird Brains’, ‘Black Mamba’ (type of snake), ‘Wacky Pony’, and ‘Wonder Worm’. The flavor ‘Roar’ evokes images of an animal.

### Concept flavor names that include terminology associated with science/technology

Our sample included some concept flavor names that were classified into the theme of science/technology or product specifications. Examples include ‘Fusion’, ‘Horsepower’, and ‘Beta’.

### Concept flavor name construction practices

Some concept flavor names used naming practices that compounded or abbreviated words or invented new words.

*Portmanteaux*

Some flavor names appear to be a portmanteau, a word created by combining two or more words together to make up another word that expresses some combination of the meaning of its parts. The flavor ‘Grappleberry’ is a combination of grape, apple and berry. ‘Ohmgurt’ is likely a combination of the words ohm (measure of electrical resistance) and yogurt. Resistance determines how much power is drawn from the battery and how much heat is generated, directly affecting the vaping experience.

*Intentional misspelling or satiric misspelling*

Sensational spelling is a marketing strategy that involves deliberately misspelling words to create a special effect. It is often used for brand names to make them more memorable and differentiate them from competitors (such as Lyft or Tumblr)^13^. The sample included many examples of this practice including flavor names ‘ez duz it’ (easy does it), ‘Fosterz’ (Fosters) and ‘krunchy squares’ (crunchy squares).

*Malapropism*

Some flavor names are types of malapropisms, puns that use the similarity of sound between two words that have different meanings. Examples include ‘ohm sweet ohm’ as a play on home-sweet-home, or ‘Bluetiful Disaster’, as a play on ‘Beautiful disaster’ but using the color blue.

### Not classified

There were 21 flavor names that were not classified into a theme and had not been created using one of the identified naming practices. These included names such as ‘Breaker’, ‘the Shape’, ‘More Please’, and ‘?Mystery?’.

## DISCUSSION

This study identified different themes and naming practices for ENDS concept flavors present in a sample of English advertisements. Some of the concept flavor names examined incorporated a variety of naming conventions often seen in other commercial products and brands, such as the use of places, people, and invented words^14^. Other concept flavor names in this sample also used terms that may evoke a sensory experience, including words suggestive of food, drink, other nicotine products, and other drugs, while other names did not include any terms that would evoke a sensory experience.

Findings from this study add to the burgeoning literature on concept flavor naming conventions. Laestadius et al.^11^ identified and classified e-liquid concept names from the Premarket Tobacco Product Applications submitted to the FDA by tobacco manufacturers producing the product (access to the list of deemed new tobacco products with timely applications reviewed by Laestadius et al.^11^ is available as of 1 April 2025 at https://www.fda.gov/tobacco-products/market-and-distribute-tobacco-product/deemed-new-tobacco-product-applications-lists). The flavor names in that sample were submitted for review and those products may or may not have entered the marketplace in the US. The flavor themes identified in this article are similar to the flavor name themes and subthemes identified by Laestadius et al.^11^ including the use of colors, drinking/drugs/altered mental states, ice and coolness, sex and romance, identities, locations, space, mythology/legend, science and technology, animals, and pop culture and media. While the flavor names in that sample may not have entered the US market (if denied authorization), findings – along with the results of the current study of concept flavors present in ENDS ads – indicate common strategies for concept flavors names that tobacco companies may use.

Importantly, the current study identified additional themes not included in the current typology scheme, including flavor names suggestive of food or drink, flavor names that include terminology associated with violence or destruction, vacation, and tropical names. This study also identified some name construction practices including the creation of portmanteaux, use of satirical or intentional misspelling, and the use of malapropisms. These themes and practices can be considered as additions to expand and refine the existing Laestadius et al.^11^ typology. Many flavor names were also classified into multiple themes, which suggests the power of concept flavor names to hold multiple meanings and levels of understanding that could appeal to a range of consumers.

### Limitations

This study has some limitations. Our sample was limited to ads in English. The sample of ads from Numerator included all ENDS ads from their monitoring efforts; however, this sample likely missed some ads, in particular from online sources. The study team used their own interpretation of concept flavor names to identify naming themes and practices and these findings may or may not align with the true intentions of the manufacturers of these products.

## CONCLUSIONS

The tobacco industry uses concept flavor names, and these names are presented in advertising in the US. This study identified some themes as well as naming practices. Future work can explore how concept flavors are interpreted by users and non-users, including how names with multiple meanings are interpreted and how names may interact with other ad content such as imagery. In addition, future work can seek to understand how concept flavor names are associated with perceived risk and how they may influence intentions to try ENDS.

## Data Availability

The data supporting this research are available from the authors on reasonable request.
